# Solid pseudopapillary tumor: a rare neoplasm of the pancreas

**DOI:** 10.1093/gastro/gou006

**Published:** 2014-02-28

**Authors:** Asim Shuja, Khalid A. Alkimawi

**Affiliations:** ^1^Department of Medicine, St. Elizabeth’s Medical Center, Brighton, MA, USA and ^2^Department of Gastroenterology, Tufts Medical Center, Boston, MA, USA

**Keywords:** Pancreas, pseudopapillary tumor, abdominal pain

## Abstract

Solid pseudopapillary tumor is a rare primary neoplasm of the pancreas that typically affects young women. It is a relatively a benign tumor, with a favorable prognosis. We here report a 27-year-old woman with solid pseudopapillary neoplasm, who presented with mild jaundice, mildly elevated liver function tests and right upper quadrant pain. Ultrasound was suggestive of hemorrhagic hepatic adenoma; however, on magnetic resonance imaging, a heterogenous mass was found in the head of pancreas. Endoscopic ultrasound-guided fine needle aspiration (FNA) revealed tumor cells with papillary architecture and immunohistochemical analysis showing cells positive for markers including beta-catenin, vimentin, alpha 1 antitrypsin etc. These findings were consistent with solid pseudopapillary neoplasm. The patient underwent pancreaticoduodenectomy. Post-surgical biopsy confirmed the FNA findings with tumor localized to the pancreas. The patient was not given any adjuvant therapy. She remained asymptomatic and showed no signs of disease after four months follow-up. It is important to differentiate this tumor from other pancreatic neoplasms, because this type is amenable to cure after complete surgical resection, even in cases with capsular invasion, unlike malignant tumors of the pancreas.

## INTRODUCTION

Solid pseudopapillary tumor (SPT) of the pancreas is a rare neoplasm, usually characterized by a well encapsulated mass, with low malignant potential. It occurs predominantly in young females, in their third decade of life. Histogenesis remains a debatable question: acinar, endocrine, ductal and progenitor cells have been postulated as possible beginnings of this tumor. It is frequently asymptomatic or minimally symptomatic [1]. These tumors can be visualized in many imaging modalities, such as ultrasonography (US), computed tomography (CT) and magnetic resonance imaging (MRI), which can be used to differentiate it from other pancreatic lesions. Complete resection is curative in most cases. In this article, we present an unusual presentation of SPT in a young female with a benign clinical course.

## CASE REPORT

A 27-year-old African American woman with no prior medical history presented with complaints of epigastric and right upper quadrant (RUQ) pain for four days. The pain had a sudden onset, with intermittent attacks; it was stabbing in nature and radiating to her back. It was associated with nausea and vomiting. There was no precipitating, relieving or aggravating factor. Review of systems was otherwise unremarkable. Her vital signs were stable. On physical examination, she was mildly icteric, her abdomen was soft, non-tender and non-distended. There were no palpable masses, and bowel sounds were audible. Laboratory data revealed aspartate transaminase (AST)-286 IU/L (*n* = 15–41), alanine transaminase (ALT)-486 IU/L (*n=*14–54), alkaline phosphatase (ALP)-94 (*n=*33–116), total bilirubin-2.8 (*n=*0.2–1.5) with direct bilirubin-1.7 (*n=*0.1–0.5). Amylase, lipase and white blood cells (WBCs) were within normal limits.

**Figure 1.**
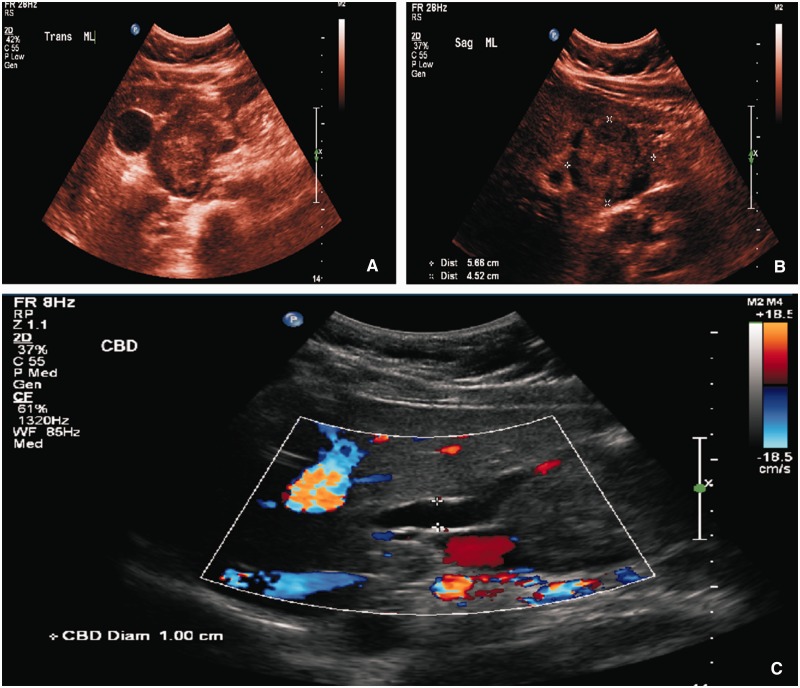
(A) Heterogenous mass at the junction of the pancreatic head & caudate lobe of the liver; (B) mass measuring 5.6 × 4.5 cm.; (C) dilated CBD-1 cm with no filling defect.

**Figure 2.**
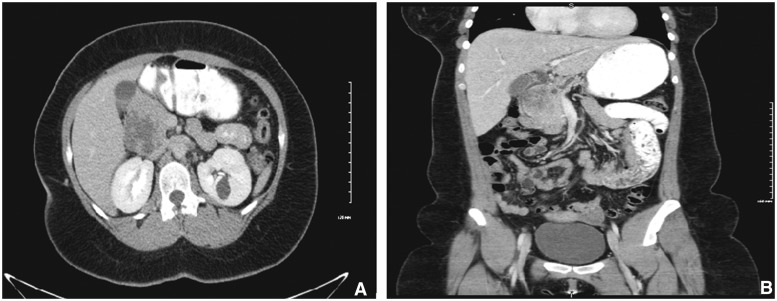
CT Abdomen and Pelvis (A) with and (B) without contrast showing pancreatic mass.

**Figure 3.**
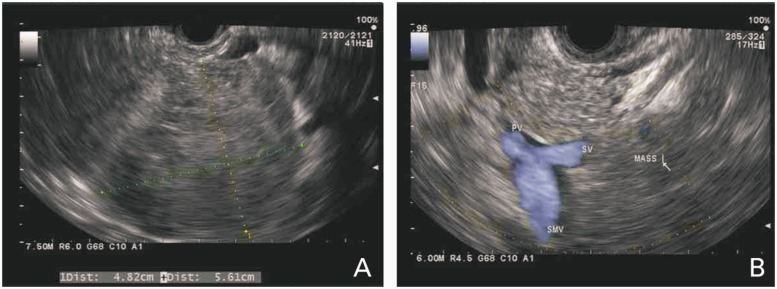
(A) EUS showing head of pancreas: solid mass; (B) pancreatic mass with possible invasion of SMV, SV and PV.

**Figure 4.**
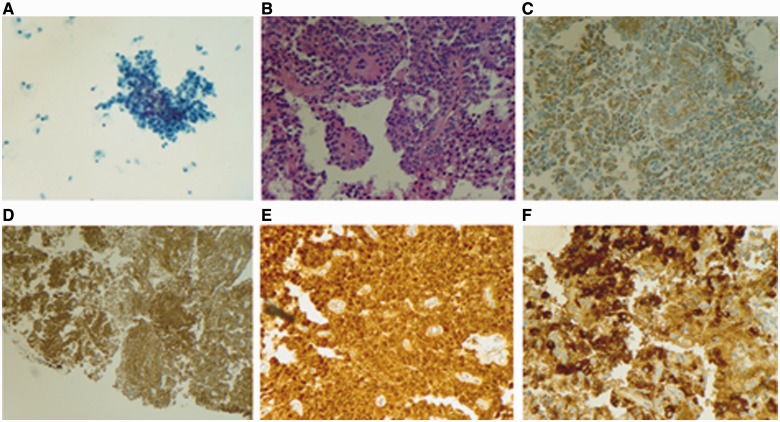
(A) Cytological examination showing cluster of tumor cells with bland nuclei; (B) pseudopapilla (HE staining); (C) vimentin positive; (D) immunostaining with CD-56 showing positivity; (E) diffuse nuclear positive staining for beta catenin; (F) alpha 1 anti-trypsin stain positive.

RUQ ultrasound (Figure 1) showed a heterogeneous, slightly echogenic and vascular ovoid mass, measuring 5.6 × 4.5 cm, situated at the junction of the pancreatic head and caudate lobe of the liver. It was suggestive of hemorrhagic hepatic adenoma. CT scan of the abdomen and pelvis (Figure 2) revealed a similar mass in the head of the pancreas, with slight dilatation of the pancreatic duct (PD) and common bile duct (CBD), having a mass-effect on the second part of the duodenum (D2). Esophago-gastro-duodenoscopy (EGD) with endoscopic ultrasound (EUS) showed a solid pancreatic head mass (4.8 × 5.6 cm), invading the portal vein (PV), superior mesenteric vein (SMV) and splenic vein (SV) (Figure 3). Fine needle aspiration (FNA) showed tumor cells with papillary architecture. An immunohistochemical analysis revealed that the tumor cells were positive for beta-catenin, vimentin, CD10, cyclin D1, CD56, progesterone receptor, alpha 1 antitrypsin (Figure 4). These findings were consistent with solid pseudopapillary neoplasm.

**Figure 5.**
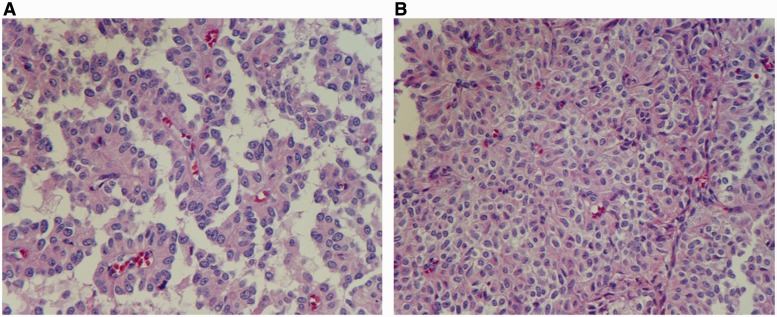
Histopathology revealing: (A) pseudopapillary cores; (B) solid, highly vascular architecture.

The patient underwent pancreaticoduodenectomy and did well post-operatively. Histological examination demonstrated a solid and vascular pattern with pseudopapillary cores (Figure 5). The tumor was found to be confined to the pancreas, with involvement of the intrapancreatic duct. There was no margin involvement. The histology also revealed perineural and probable angiolymphatic invasion. Almost four days after the surgery, the patient’s AST and ALT trended down to 52 IU/L and 36 IU/L, respectively, and she denied any abdominal pain. Given the concern of neurovascular involvement, the patient was enrolled into an imaging surveillance program. She has been asymptomatic, not requiring any adjunctive therapy.

## DISCUSSION

Solid pseudopapillary tumor (SPT) of the pancreas is a rare exocrine pancreatic tumor, which represents only about 1% of all tumors of the pancreas. It was first described by Frantz in 1959. Different names for this tumor were reported until it was defined by the World Health Organization (WHO) in 1996 as a “solid pseudopapillary tumor” of the pancreas [2]. The origin of solid pseudopapillary tumors is unclear. Many investigators favor the theory that SPTs originate from multipotent primordial cells, whereas others suggest an extrapancreatic origin, from genital ridge angle-related cells [3]. This rare tumor seems to have a predilection for young Asian and African-American women. The male to female ratio is 1:10 and the mean age at presentation is 22 years. It is often clinically asymptomatic or present with a gradually enlarging abdominal mass. Jaundice is a rare presentation [4]. Although most SPTs exhibit benign behavior, malignancy can occur in about 15% of cases, manifesting as metastases or invasion of adjacent structures [3]. Reported studies indicated that the most common metastatic sites were the liver and the omentum [5, 6]. The majority of such tumors are located in the pancreatic body and tail. The morphological appearance of SPT varies from solid to cystic components with cellular degenerative changes [7]. They are characteristically positive for α*1*-antitrypsin, CD56, CD10, and vimentin [3]. The majority of tumors are diagnosed through ultrasound or CT scan of the abdomen, but MRI also defines the hypervascular, well-encapsulated, round tumors with mixed cystic and solid components. Echo-endosonography may provide FNA biopsy with the possibility of pre-operative pathologic diagnosis [3]. Despite the locally aggressive features, the tumor has a low-grade malignant potential and tends to have a favorable prognosis, even in the presence of metastatic disease. Overall 5-year survival is as high as 97% in patients undergoing surgical resection [8]. Neither vascular, or perineural invasion has been a factor for predicting tumor recurrence or overall survival of patients [4]. Surgery is the treatment of choice, even in the case of distant hepatic metastasis or local recurrence [9].

In conclusion, pancreatic pseudopapillary tumors are rare neoplasms with malignant potential. They may present as an abdominal mass, abdominal pain or, rarely, with jaundice, as in this case. Timely resection on diagnosis provides long-term survival.

**Conflict of interest:** none declared.

## References

[1] Oliveira Lima S, Rocha Santana V, Correia Leao S (2012). Solid-pseudopapillary tumor of pancreas in a young woman: a case report and literature review. Rev Med Chil.

[2] Coleman KM, Doherty MC, Bigler SA (2003). Solid pseudopapillary tumor of the pancreas. Radiographics.

[3] Zuriarrain A, Nir I, Bocklage T (2011). Pseudopapillary tumor of the pancreas in a 17–year-old girl. J Clin Oncol.

[4] Chen X, Zhou GW, Zhou HJ (2005). Diagnosis and treatment of solid-pseudopapillary tumors of the pancreas. Hepatobiliary Pancreas Dis Int.

[5] Sun CD, Lee WJ, Choi JS (2005). Solid-pseudopapillary tumours of the pancreas: 14 years' experience. ANZ J Surg.

[6] Mao C, Guvendi M, Domenico DR (1995). Papillary cystic and solid tumors of the pancreas: a pancreatic embryonic tumor? Studies of three cases and cumulative review of the world's literature. Surgery.

[7] Bardales RH, Centeno B, Mallery JS (2004). Endoscopic ultrasound-guided fine-needle aspiration cytology diagnosis of solid-pseudopapillary tumor of the pancreas: a rare neoplasm of elusive origin but characteristic cytomorphologic features. Am J Clin Pathol.

[8] Eder F, Schulz HU, Rocken C (2005). Solid-pseudopapillary tumor of the pancreatic tail. World J Gastroenterol.

[9] Mulkeen AL, Yoo PS, Cha C (2006). Less common neoplasms of the pancreas. World J Gastroenterol.

